# Dual Role of microRNA-146a in Experimental Inflammation in Human Pulmonary Epithelial and Immune Cells and Expression in Inflammatory Lung Diseases

**DOI:** 10.3390/ijms25147686

**Published:** 2024-07-13

**Authors:** Lucia Gronau, Ruth P. Duecker, Silvija-Pera Jerkic, Olaf Eickmeier, Jordis Trischler, Andreas G. Chiocchetti, Katharina Blumchen, Stefan Zielen, Ralf Schubert

**Affiliations:** 1 Department of Pediatrics, Division of Pneumology, Allergology, Infectious Diseases and Gastroenterology, University Hospital, Goethe University Frankfurt, 60590 Frankfurt am Main, Germany; gronau@med.uni-frankfurt.de (L.G.); duecker@med.uni-frankfurt.de (R.P.D.); pera.silvija.jerkic@gmail.com (S.-P.J.); eickmeier@med.uni-frankfurt.de (O.E.); trischler@med.uni-frankfurt.de (J.T.); bluemchen@med.uni-frankfurt.de (K.B.); 2 Department of Child and Adolescent Psychiatry, Psychosomatics and Psychotherapy, University Hospital, Goethe University Frankfurt, 60590 Frankfurt am Main, Germany; andreas.chiocchetti@med.uni-frankfurt.de; 3 Respiratory Research Institute, Medaimun GmbH, 60596 Frankfurt am Main, Germany; s.zielen@medaimun.de

**Keywords:** microRNA, inflammation, lung disease, miR-146a, isomiRs

## Abstract

microRNA (miR)-146a emerges as a promising post-transcriptional regulator in various inflammatory diseases with different roles for the two isoforms miR-146a-5p and miR-146a-3p. The present study aimed to examine the dual role of miR-146a-5p and miR-146a 3p in the modulation of inflammation in human pulmonary epithelial and immune cells in vitro as well as their expression in patients with inflammatory lung diseases. Experimental inflammation in human A549, HL60, and THP1 via the NF-kB pathway resulted in the major upregulation of miR-146a-5p and miR-146a-3p expression, which was partly cell-specific. Modulation by transfection with miRNA mimics and inhibitors demonstrated an anti-inflammatory effect of miR-146a-5p and a pro-inflammatory effect of miR-146a-3p, respectively. A mutual interference between miR-146a-5p and miR-146a-3p was observed, with miR-146a-5p exerting a predominant influence. In vivo NGS analyses revealed an upregulation of miR-146a-3p in the blood of patients with cystic fibrosis and bronchiolitis obliterans, while miR-146a-5p levels were downregulated or unchanged compared to controls. The reverse pattern was observed in patients with SARS-CoV-2 infection. In conclusion, miR-146a-5p and miR-146a-3p are two distinct but interconnected miRNA isoforms with opposing functions in inflammation regulation. Understanding their interaction provides important insights into the progression and persistence of inflammatory lung diseases and might provide potential therapeutic options.

## 1. Introduction

Inflammatory lung diseases rank among the leading causes of global mortality [1], with chronic airway inflammation serving as a major cause of pathophysiology in various respiratory diseases, including chronic obstructive pulmonary disease (COPD) [2], cystic fibrosis (CF) [3], asthma [4], acute respiratory distress syndrome (ARDS) [5], and bronchiolitis obliterans (BO) [6,7]. The inflammatory response itself is a beneficial and indispensable process. However, maintaining homeostasis between pro- and anti-inflammatory mediators is paramount to prevent tissue damage. An imbalance or lack of resolution can lead to the establishment of chronic inflammation within the tissue. The infiltration of inflammatory cells such as neutrophilic granulocytes, macrophages, and lymphocytes in the airways and lung tissue is common [2,8]. The continual release of pro-inflammatory cytokines from these cells perpetuates inflammation, ultimately contributing to airway remodeling, fibrosis, and the development of chronic lung diseases [2,9].

The treatment options for respiratory diseases are most commonly limited to the reduction in symptoms with a primary focus on diminishing inflammation to prevent and alleviate exacerbations [10]. Corticosteroids, commonly employed as first-line therapy, exhibit efficiency in conditions such as asthma, yet their effectiveness is limited in COPD, CF, and BO [10,11,12]. Monoclonal antibodies, targeting pro-inflammatory cytokines like TNF-α or CXCL-8 in COPD, are currently under the focus of research. However, their high cost and inconsistent superiority compared to standard treatment pose challenges [13,14]. These limitations underscore the imperative need for developing new treatment strategies for chronic airway inflammation.

In 1993, a new field of RNA research was discovered, focusing on the application of microRNAs (miRNAs). The small, non-coding RNAs, approximately 22 nucleotides in length, are highly conserved and play a crucial role in post-transcriptional gene regulation [15]. miRNAs are transcribed from exons or introns in the nucleus as a precursor molecule that forms a hairpin structure and is then processed into a miRNA duplex in the cytoplasm. The two strands of the miRNA duplex are called the guide strand and the passenger strand, with the guide strand being the functional strand that binds to the target mRNAs, while the passenger strand is normally degraded. When miRNAs are incorporated into the RNA-induced silencing complex (RISC), they can bind to complementary sequences on target mRNA molecules. This binding leads to the inhibition of the translation or degradation of the target mRNA, which ultimately results in reduced protein expression. Each miRNA has a specific number of target genes that it can regulate [16]. miRNA dysregulation has been associated with diseases like cardiovascular diseases [17], cancer [18], and autoimmune [19] or respiratory diseases [20]. This observation opens promising avenues for novel therapeutic targets with ongoing clinical studies already underway [21,22].

Among the extensively studied miRNAs, miR-146a stands out as a pivotal regulator of the immune system. The dysregulation of miR-146a has been described in various lung diseases, including COPD [23], post-infectious bronchiolitis obliterans (PiBO) [24], asthma [25], and COVID-19 [26]. Present in numerous immune cell types, miR-146a serves as a critical negative feedback regulator of the NF-kB pathway during inflammation [27,28]. Notably, miR-146a produces two mature strands, namely miR-146a-5p and miR-146a-3p. During the miRNA arm selection, a guide strand is predominantly selected for binding to the RISC complex, facilitating miRNA silencing or degrading, while the passenger strand is typically degraded. However, in some instances, both mature strands can be functional, influencing gene expression [29].

The guide strand, miR-146a-5p, is known to have anti-inflammatory effects through the downregulation of Toll-like receptor (TLR) and cytokine receptor signaling, directly targeting interleukin-1 receptor-associated kinase 1 (*IRAK1*) and tumor necrosis factor receptor-associated factor 6 (*TRAF6*) [27,30]. Its expression is upregulated in response to inflammatory stimuli, serving as a natural control mechanism for inflammation [31]. Conversely, the downregulation of miR-146a-5p is often associated with a more pro-inflammatory phenotype, fibrosis or chronic inflammation, e.g., in COPD and bronchitis [23,32,33]. In contrast, the passenger strand, miR-146a-3p, is less characterized. The reduction in miR-146a-3p expression levels is linked to beneficial effects, whereas significantly increased miR-146a-3p levels are often observed in disease-affected organs [34,35].

However, the consequences of dysregulated miRNA expression can vary significantly depending upon the cell type, tissue, or underlying disease [36,37]. Notably, isomiRs, representing two mature miRNAs deriving from the same pre-miRNA, can exert synergistic and antagonistic effects within a single cell [38,39]. This demonstrates the great importance of considering both arms of a relevant miRNA simultaneously. Despite the acknowledged significance, only a limited number of publications have addressed miR-146a-5p and miR-146a-3p as a central research question [20,40,41].

Consequently, this study aims to explore the expression, function, and interaction of miR-146a-5p and -3p comprehensively in the context of inflammation. For this, the primary inflammatory cells implicated in chronic respiratory diseases were investigated using a cell culture model to induce experimental inflammation. Airway epithelial cells (A549), neutrophil-precursor cells (HL60), and macrophages (THP1) were stimulated with pro-inflammatory cytokines to induce inflammation. The NF-kB-driven modulation of miR-146a expression in these cell models has already been shown previously [27,30,42,43]. Results of the analyzed cells showed a crucial anti-inflammatory role of miR-146a-5p in all three cell lines and pro-inflammatory properties of miR-146a-3p in A549 and THP1 cells with the mutual interaction of the isomiRs. Additionally, their expression was analyzed in patients with PiBO, CF, and COVID-19 as representative examples for inflammatory lung diseases.

## 2. Results

### 2.1. In Vitro Stimulation of Lung Epithelial Cells, M1 Macrophages, and Neutrophil-Precursor Cells Induces Activation of NF-kB Pathway

**Figure 1:**
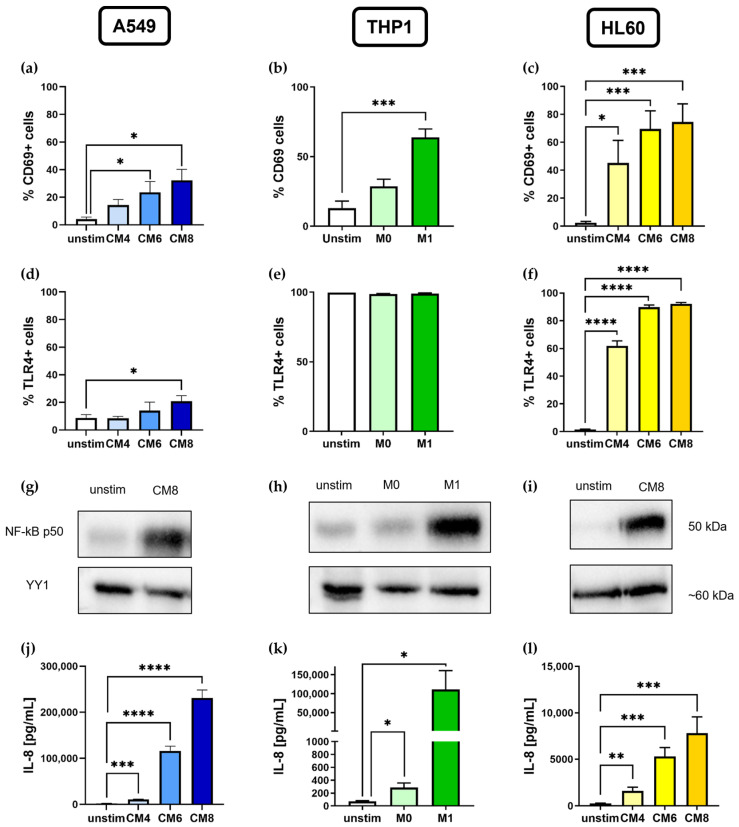
The in vitro stimulation of lung epithelial cells, macrophages, and neutrophil-precursor cells. Depicted are flow cytometric analyses showing CD69+ (**a**–**c**)- and TLR4+ (**d**–**f**)-stimulated A549, THP1 and HL60 cells. The activation of the NF-kB pathway was detected via a Western blot in all three cell lines, showing the comparison of the NF-kB p50 band in unstimulated and stimulated cells at 50 kDa. YY1 is used as an endogenous control for nuclear protein expression (**g**–**i**). Further, IL-8 protein expression levels were measured by a cytometric bead array (CBA) after cytokine mix (CM4, CM6, and CM8) stimulation in A549 (**j**) and in HL60 cells (**l**), as well as after M1 differentiation in THP1 cells (**k**). A549: *n* = 4; THP1: *n* = 3–8; HL60: *n* = 3–7. * *p* < 0.05, ** *p* < 0.01, *** *p* < 0.001, **** *p* < 0.0001.

To establish an experimental inflammatory system in vitro, A549 and HL60 were stimulated with different concentrations of a cytokine mixture (CM4, CM6, CM8) containing IL-1β, INF-γ, and TNF-α, whereas THP1 cells were differentiated into M1 macrophages using LPS and IFN-γ. Experimental inflammation led to the activation of A549, THP1, and HL60 cells (Figure 1). The expression of the activation marker CD69 was measured by flow cytometry showing increased CD69+ cells after stimulation in a dose-dependent manner in all three cell lines (A549: unstim: 4.2 ± 1.4%, CM6: 23.6 ± 7.8%, *p* = 0.0499; CM8: 32.2 ± 8.1%, *p* = 0.014, Figure 1a; THP1: unstim: 13.1 ± 5.0%, M1: 63.9 ± 6.1%, *p* = 0.0002, Figure 1b; HL60: unstim: 2.4 ± 1.1%, CM4: 45.3 ± 16.2%, *p* = 0.0299; CM6: 69.5 ± 13.1%, *p* = 0.0009; CM8: 74.6 ± 12.9%, *p* = 0.0005, Figure 1c). Further, cell-specific markers were upregulated after stimulation, including CD40 on CM6- and CM8-stimulated A549 cells (Figure S1a), CD80 and CD86 on M1 THP1 cells (Figure S1b,c), and CD11b on CM4-, CM6-, and CM8-stimulated HL60 cells (Figure S1d).

To prove NF-kB-specific activation, TLR4 expression as well as nuclear NF-κB p50 levels were analyzed. TLR4+-stimulated A549 and HL60 cells were found to be significantly upregulated compared to unstimulated cells (A549: unstim: 8.7 ± 2.5%, CM8: 20.9 ± 4.0%, *p* = 0.041, Figure 1d; HL60: unstim: 1.4 ± 0.4%, CM4: 61.9 ± 3.6%, CM6: 89.8 ± 1.5%, CM8: 92.2 ± 1.0%, all *p* < 0.0001, Figure 1f), while almost 100% of the THP1 cells already expressed TLR4 on their surface even in an unstimulated state (Figure 1e). NF-kB activation was demonstrated by a Western blot detecting a strong NF-kB p50 band in all three cell lines after stimulation with CM8 or LPS and IFN-γ, confirming the activation of the NF-kB pathway (Figure 1g–i).

Stimulation also resulted in increased IL-8 concentrations in the cell culture supernatants of A549 cells (unstim: 2105 ± 592.3 pg/mL, CM4: 10,755 ± 798.2 pg/mL; *p* = 0.0001; CM6: 116,350 ± 9968 pg/mL, *p* < 0.0001; CM8: 231,247 ± 17,126 pg/mL, *p* < 0.0001, Figure 1j), of HL-60 cells (unstim: 255.6 ± 39.8 pg/mL, CM4: 1604 ± 404.5 pg/mL; *p* = 0.006; CM6: 5322 ± 946.1 pg/mL, *p* = 0.0002, CM8: 7824 ± 1742 pg/mL, *p* = 0.001, Figure 1l), and of differentiated M1 macrophages (unstim: 73.9 ± 9.4 pg/mL, M0: 285.6 ± 72.0 pg/mL, *p* = 0.015; M1: 111,105 ± 49,522 pg/mL, *p* = 0.049; Figure 1k).

In addition to IL-8, there was also a significant increase in IL-6 expression in A549 cells (Figure S1e). However, IL-6 could only be detected in A549 and THP1 cells. Therefore, IL-8 was used as a marker for the successful stimulation of the cells in further experiments. Additionally, CM8 was regarded as an ideal stimulation concentration in A549 and HL60 cells for further experiments.

### 2.2. miR-146a-5p Is the Most Exclusively Upregulated miRNA in Stimulated A549, THP1, and HL60 Cells

**Figure 2:**
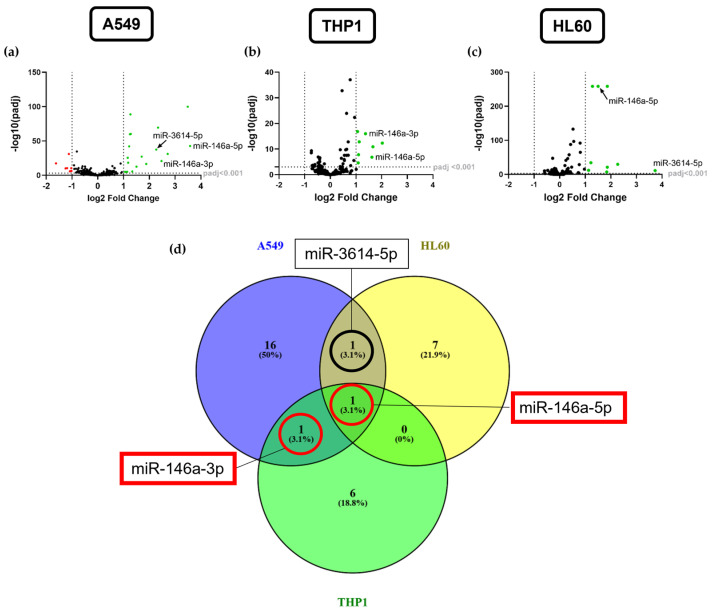
Analysis of miRNA expression in stimulated cell lines. Volcano plots illustrate differentially expressed miRNAs measured by next generation sequencing (NGS) in CM8-stimulated A549 cells (**a**), M1 THP1 cells (**b**), and CM8-stimulated HL60 cells (**c**) compared to unstimulated or M0 THP1 cells, respectively (*n* = 4). Green dots represent significantly upregulated miRNAs (log2 fold change (FC) > 1; padj < 0.001) and red dots represent significant downregulation of miRNA expression (log2 FC < −1; padj < 0.001). Vertical dotted lines in the volcano plots demonstrate the thresholds of fold changes for further analyses. Venn blot analysis (Venny 2.1) shows comparison of significantly upregulated miRNAs with padj < 0.001 and log2 FC > 1 in stimulated A549, THP1, and HL60 cells (**d**).

The cell lines were analyzed for differentially expressed global miRNAs in stimulated samples compared to unstimulated controls. The next generation sequencing (NGS) analysis of A549 cells revealed 17.8 Mio total reads and an average of 2 Mio miRNA reads per sample. A total of 1488 miRNAs were detected of which 578 had five or more reads in each sample. A total of 114 differentially expressed miRNAs were identified that passed a threshold of adjusted *p* value (padj) < 0.001 of which 62 were significantly upregulated and 52 downregulated. Of those, 19 miRNAs were significantly upregulated with a fold change (FC) >2 and 8 miRNAs with a fold change <−2 were significantly downregulated (Figure 2a, Table S1).

For THP1 cells, the total number of reads was 10.6 Mio averaging to 1.3 Mio reads per sample. A total of 452 miRNAs were detected overall and of those, 322 had five or more reads in each sample. A total of 46 miRNAs were expressed differentially with padj < 0.001 in M1 differentiated THP1 cells compared to M0 cells of which 27 were upregulated and 19 downregulated. Eight significantly upregulated miRNAs were detected, which exhibited a fold change >2 (Figure 2b, Table S1).

HL60 cells showed a total of 8.4 Mio reads with an average of 1.1 Mio miRNA reads per sample and revealed 432 detected miRNAs of which 355 had five or more reads in each sample. A total of 60 miRNAs revealed significantly differential expression below padj < 0.001 of which 37 miRNAs were upregulated and 23 downregulated. Nine significantly upregulated miRNAs showed a fold change >2 compared to unstimulated cells and were selected for a further analysis (Figure 2c, Table S1).

miRNAs with padj < 0.001 and fold change >2 were selected from the differential expression analysis for a further analysis and were compared for intersections between the three cell lines as shown in the Venn diagram (Figure 2d).

The analysis demonstrated that miR-146a-5p is the only significantly increased miRNA expressed in all three cell lines after stimulation via the NF-κB pathway. Interestingly, its counterpart miR-146a-3p is significantly upregulated in stimulated A549 and THP1 cells but was not found in stimulated HL60 cells. miR-3614-5p was found to be a common miRNA of the stimulated A549 and HL60 cells, whereas no overlapping miRNA of the HL60 and THP1 cells was found that met the above criteria. Furthermore, cell-specific miRNAs were found for A549 (*n* = 16), HL60 (*n* = 7), and THP1 cells (*n* = 6). The expression of miR-146a-5p, miR-146a-3p, and miR-3614-5p was validated by qPCR in all three cell lines (Figure S2).

### 2.3. Transfection with miR-146a-5p Mimic as Well as Anti-miR-146a-3p Results in Cell-Specific Downregulation of IL-8 Expression Levels

**Figure 3:**
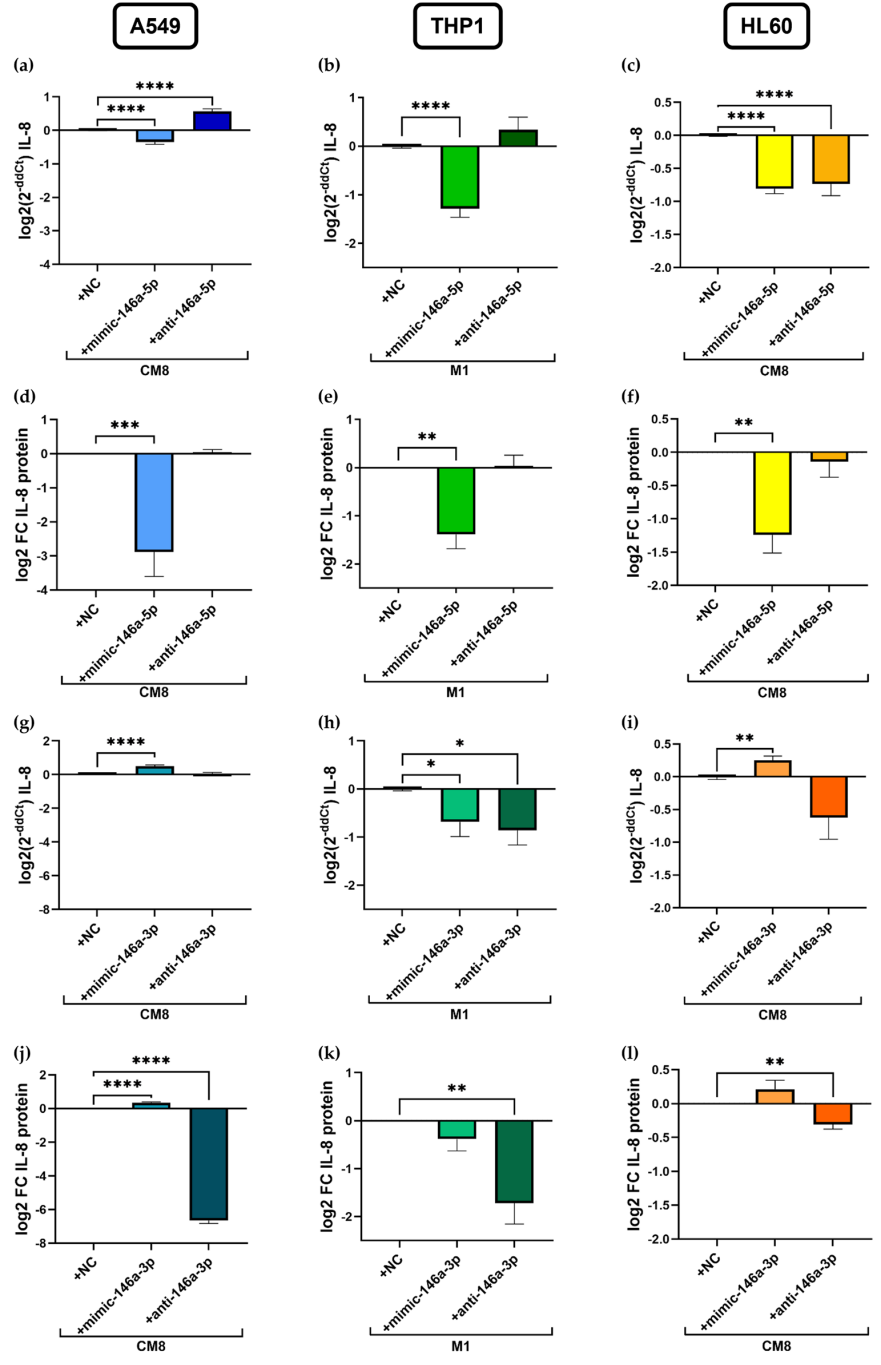
Effects of miRNA transfection on *IL-8* mRNA and protein expression. Stimulated A549, THP1, and HL60 cells were transfected with mimics and inhibitors of miR-146a-5p (**a**–**f**) and miR-146a-3p (**g**–**l**). *IL-8* mRNA expression was measured by qPCR in CM8-stimulated A549 cells (**a**,**g**), in M1 THP1 cells (**b**,**h**), and in CM8-stimulated HL60 cells (**c**,**i**). Protein expression was measured by CBA in supernatants of cell culture samples (**d**–**f**,**j**–**l**). FC = fold change; NC = negative control. A549: *n* = 6–12, THP1: *n* = 3–5, HL60: *n* = 3–6, * *p* < 0.05, ** *p* < 0.01, *** *p* < 0.001, **** *p* < 0.0001.

Furthermore, the modulatory effect of miR-146a-5p and of miR-146a-3p on inflammation was investigated using miRNA mimics and inhibitors (Figure 3). At this point, it is worthwhile to mention that highly increased miRNA levels were detected after mimic transfection, whereas not in all cell lines a significant downregulation of expression levels was observed after inhibitor transfection due to the methodology (Figure S3). miRNA expression levels do not accurately report the level of functional miRNA, which is especially true for antisense inhibitor levels. In addition, qPCR is not a suitable method to measure inhibitors [44], so we did functional experiments with a control gene. However, the functional effects of transfection with miR-146a mimics and inhibitors on experimental inflammation in A549, THP1, and HL60 cells were assessed by measuring levels of *IL-8* mRNA and IL-8 protein by qPCR and cytometric bead array (CBA), respectively.

The transfection of stimulated cells with the miR-146a-5p mimic resulted in a significant decrease in *IL-8* mRNA as well as in IL-8 protein in A549 cells (mRNA: −0.35 ± 0.07 log2(2^−ddCt^), *p* < 0.0001, Figure 3a; protein: -2.9 ± 0.7 log2 FC, *p* = 0.0006, Figure 3d), in HL60 cells (mRNA: −0.8 ± 0.07 log2(2^−ddCt^), *p* < 0.0001, Figure 3c; protein: −1.2 ± 0.3 log2 FC, *p* = 0.0019, Figure 3f), and in THP1 cells (mRNA: −1.3 ± 0.2 log2(2^−ddCt^), *p* < 0.0001, Figure 3b; protein: −1.4 ± 0.3 FC, *p* = 0.0015, Figure 3e) compared to negative control- (NC) transfected cells. In contrast, transfection with the anti-miR-146a-5p significantly increased *IL-8* mRNA in A549 cells (0.6 ± 0.1 log2(2^−ddCt^); *p* < 0.0001) but reduced the expression in HL60 cells (−0.7 ± 0.2 log2(2^−ddCt^), *p* < 0.0001) without affecting the IL-8 protein levels.

Transfection with the miR-146a-3p mimic led to a significant increase in IL-8 expression at both mRNA (0.5 ± 0.1 log2(2^−ddCt^), *p* < 0.0001, Figure 3g) and protein levels (0.3 ± 0.1 log2 FC, *p* < 0.0001, Figure 3j) in A549 cells, as well as in *IL-8* mRNA levels in HL60 cells (0.3 ± 0.1 log2(2^−ddCt^), *p* = 0.007, Figure 3i), while *IL-8* mRNA expression was decreased in THP1 cells (−0.7 ± 0.3 log2(2^−ddCt^), *p* = 0.04; Figure 3h). However, reduced *IL-8* mRNA after transfection with the miR-146a-3p inhibitor was only observed in THP1 cells (−0.9 ± 0.3 log2(2^−ddCt^), *p* = 0.014), and a significant decrease in IL-8 was observed at the protein level in all three cell lines (A549: −6.7 ± 0.2 log2 FC, *p* < 0.0001, Figure 3j; THP1: −1.7 ± 0.4 log2 FC, *p* = 0.007, Figure 3k; HL60: −0.3 ± 0.1 log2 FC, *p* = 0.009, Figure 3l). Similar observations were made regarding *IL-6* mRNA and protein levels after transfection in A549 cells (Figure S4).

### 2.4. Modification of miR-146a-5p and miR-146a-3p Significantly Impacts IL-8 mRNA and Protein Expression in A549 Cells

To determine whether a combined modulation of both miRNAs can have additive effects on IL-8 expression, the miR-146a-5p mimic and anti-miR-146a-3p or anti-miR-146a-5p and miR-146a-3p mimic were applied in combination as well as separately in A549 and in THP1 cells. Since HL60 cells showed no detectable expression of miR-146a-3p, these cells were not included in the experiments.

**Figure 4:**
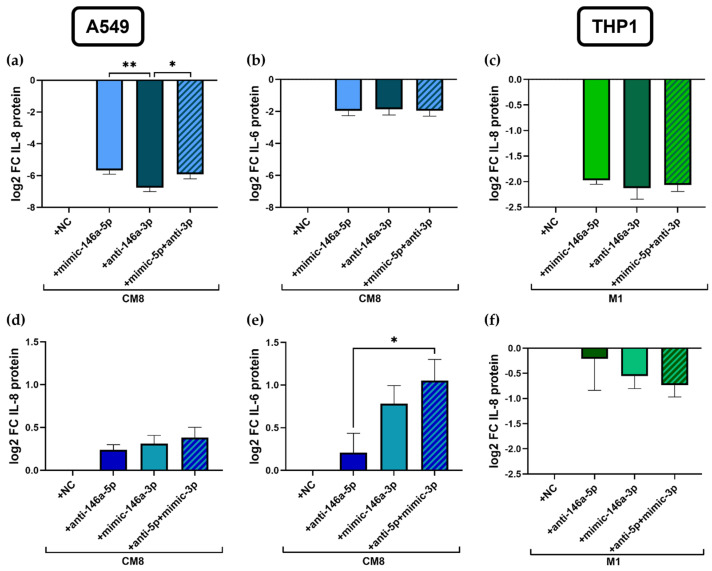
The impact of the simultaneous modification of miR-146a-5p and miR-146a-3p on cytokine expression. The mimic of miR-146a-5p and the inhibitor of miR-146a-3p were transfected separately and in combination in stimulated A549 (**a**,**b**) and THP1 cells (**c**), measuring IL-8 (**a**,**c**) and IL-6 protein expression (**b**) in the supernatant of cell culture samples. The reverse combination consisting of transfection with the miR-146a-5p inhibitor and miR-146a-3p mimics was applied in (**d**–**f**). A549: *n* = 5–6, THP1: *n* = 3; * *p* < 0.05, ** *p* < 0.01.

The direct comparison demonstrated a stronger IL-8 protein inhibition by the miR-146a-3p inhibitor compared to the miR-146a-5p mimic (mimic 146a-5p: -5.7 ± 0.2 log2 FC, anti-miR-146a-3p: −6.8 ± 0.2 log2 FC; *p* = 0.008; Figure 4a). However, the combination was less inhibitory than the anti-miR-146a-3p itself (combination: −5.9 ± 0.3 log2 FC, *p* = 0.046) and showed no difference in IL-8 regulation compared to the miR-146a-5p mimic transfection (Figure 4a). The analysis of IL-6 protein expression revealed no significant differences between the single and combined treatments (Figure 4b).

Vice versa, transfection with anti-miR-146a-5p and miR-146a-3p mimics showed no effects on IL-8 protein expression in A549 cells (Figure 4d). However, the combination of anti-miR-146a-5p and miR-146a-3p mimics led to a significant increase in IL-6 protein expression compared to anti-miR-146a-5p transfection (mimic 146a-5p: 0.8 ± 0.2 log2 FC, combination: 1.1 ± 0.2 log2 FC, *p* = 0.04; Figure 4e).

Interestingly, mRNA expression patterns of *IL-8* and *IL-6* were reversed after transfection with anti-miR-146a-3p and showed significant upregulation compared to miR-146a-5p mimic transfection and to the combinatory treatment (Figure S5a,b). Further, transfection with only the miR-146a-3p mimic and the combination of anti-146a-5p and miR-146a-3p mimics both showed the upregulation of *IL-8* mRNA compared to the treatment with only anti-miR-146a-5p in A549 cells (Figure S5d).

In M1-differentiated THP1 cells, using the same combination of the miR-146a-5p mimic and miR-146a-3p inhibitor, no difference of IL-8 protein expression was detected between the various transfections (Figure 4c). On mRNA levels, miR-146a-5p mimic transfection showed more reduced *IL-8* expression compared to the inhibitor of miR-146a-3p (Figure S5c). Conversely, combinatory transfection with anti-miR-146a-5p and miR-146a-3p mimics showed reduced *IL-8* expression compared to individual anti-miR-146a-5p transfection on mRNA (Figure S5f) but not on the protein level (Figure 4f).

### 2.5. Target Identification Analysis of miR-146a-5p and miR-146a-3p

**Figure 5:**
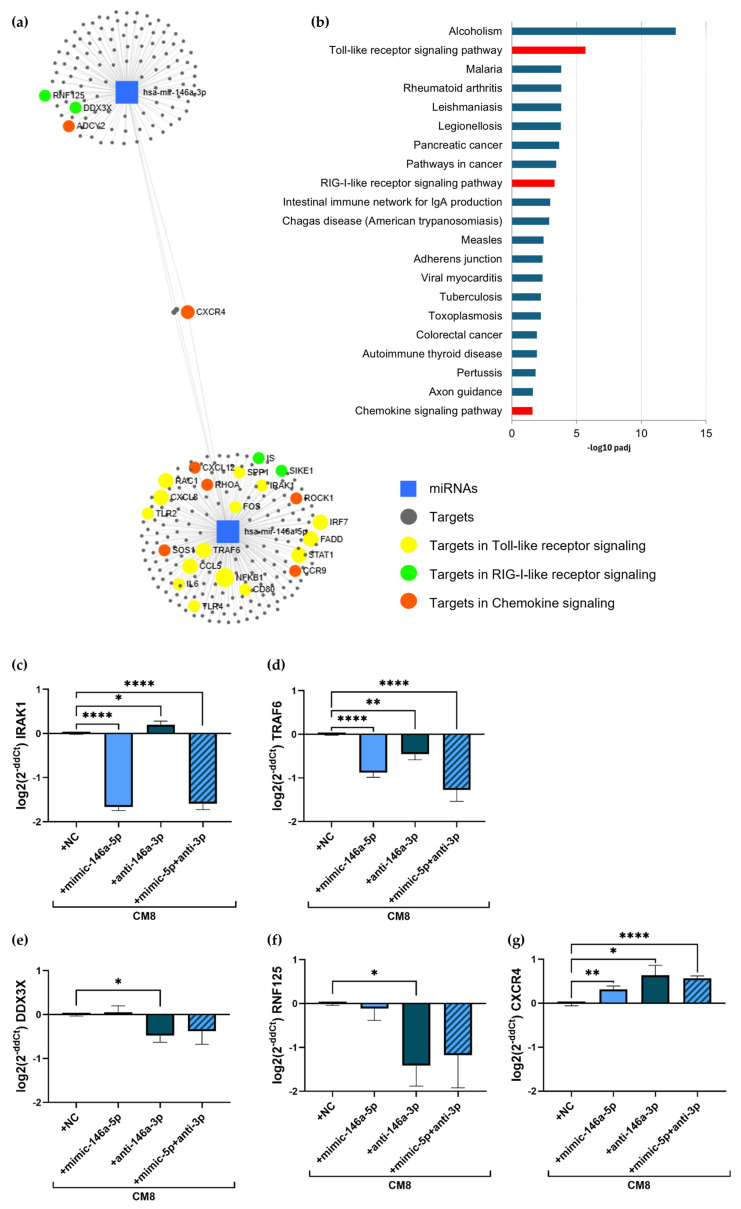
Target analysis of miR-146a-5p and miR-146a-3p. In silico target analysis was performed using www.mirnet.ca showing 203 targets of miR-146a-5p and 116 targets of miR-146a-3p including three overlapping targets. KEGG pathway analysis demonstrates 15 targets implicated in Toll-like receptor signaling (yellow), eight genes involved in RIG-I-like receptor signaling (green), and 12 targets belonging to chemokine signaling pathway (orange) (**a**). Targets that are allocated to multiple pathways are shown as increased dots. Pathways found in the KEGG pathway analysis and their respective padj values are displayed in (**b**) with relevant pathways colored in red. Effects of target modulation by miR-146a-5p mimic and anti-miR-146a-5p transfection were measured individually and in combination in stimulated A549 cells. qPCR measurements show expression of *IRAK1* (**c**), *TRAF6* (**d**), *DDX3X* (**e**), *RNF125* (**f**), and *CXCR4* (**g**) in transfected cells. *n* = 3. * *p* < 0.05, ** *p* < 0.01, **** *p* < 0.0001.

To identify further targets of miR-146a-5p and miR-146a-3p, an in silico target analysis was performed using www.mirnet.ca and genes from the database miRTarBase v8.0 (Figure 5a). In total, 203 targets of miR-146a-5p and 116 targets of miR-146a-3p including three overlapping targets were identified. The Kyoto Encyclopedia of Genes and Genomes (KEGG) pathway analysis showed 15 targets implicated in Toll-like receptor (TLR) signaling (padj = 0.00000203), eight genes allocated to RIG-I-like receptor signaling (padj = 0.000519), and 12 targets involved in the chemokine signaling pathway (padj = 0.0262, Figure 5b). Two targets from miR-146a-5p (*IRAK1*, *TRAF6*), two from miR-146a-3p (*DDX3X, RNF125*), and one shared target (*CXCR4*) were selected for the modulation analysis by qPCR. As anti-inflammatory properties of the miR-146a-5p mimic and miR-146a-3p inhibitor were observed in our experiments before, the effect of these transfections on target modulation were examined in A549 cells to confirm the target specificity. The transfection of the miR-146a-5p mimic as well as the combination of both miRNAs led to a significant downregulation of *IRAK1* (mimic 146a-5p: −1.7 ± 0.1 log2(2^−ddCt^), *p* < 0.0001; combination: −1.6 ± 0.1 log2(2^−ddCt^), *p* < 0.0001; Figure 5c) and *TRAF6* (mimic 146a-5p: 0.9 ± 0.1 log2(2^−ddCt^), *p* < 0.0001; combination: −1.3 ± 0.3 log2(2^−ddCt^), *p* = 0.0001; Figure 5d) whereas no effect was detected on *DDX3X* (Figure 5e) and *RNF125* expression (Figure 5f). In contrast, the inhibitor of miR-146a-3p increased *IRAK1* expression (0.2 ± 0.1 log2(2^−ddCt^), *p* = 0.0315), and downregulated the expression of *TRAF6* (−0.5 ± 0.1 log2(2^−ddCt^), *p* = 0.0034), *DDX3X* (−0.5 ± 0.2 log2(2^−ddCt^), *p* = 0.0108), and *RNF125* (−1.4 ± 0.5 log2(2^−ddCt^), *p* = 0.0123). However, the expression of the shared target *CXCR4* was upregulated after both single and the combinatory transfection (mimic 146a-5p: 0.3 ± 0.1 log2(2^−ddCt^), *p* = 0.0022; anti-miR-146a-5p: 0.6 ± 0.2 log2(2^−ddCt^), *p* = 0.0102; combination: 0.6 ± 0.1 log2(2^−ddCt^), *p* < 0.0001; Figure 5g).

### 2.6. miR-146a-5p Mimic Impacts miR-146a-3p Expression in Lung Epithelial Cells and in M1 Macrophages In Vitro

**Figure 6:**
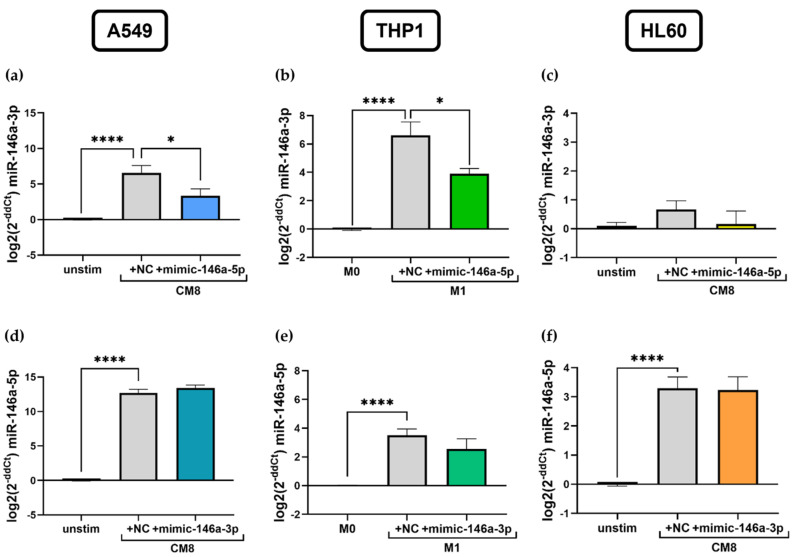
Mutual interference of miR-146a-5p and miR-146a-3p expression in vitro. miR-146a-3p expression was measured by qPCR after transfection with miR-146a-5p mimic in stimulated A549 cells (**a**), in M1 THP1 cells (**b**), and in stimulated HL60 cells (**c**). miR-146a-5p expression after transfection with miR-146a-3p mimic in the respective cell lines is depicted in (**d**–**f**). A549: *n* = 4–6, THP1: *n* = 6–7, HL60: *n* = 3–5. * *p* < 0.05, **** *p* < 0.0001.

In further experiments, a mutual interaction between miR-146a-5p and miR-146a-3p was analyzed. The expression of miR-146a-3p was measured by qPCR after miR-146a-5p mimic transfection and vice versa. Interestingly, a cell-specific regulation of miRNA expression was found. Levels of miR-146a-3p were significantly decreased in A549 cells after transfection with the miR-146a-5p mimic compared to NC-treated cells (NC: 6.6 ± 1.0 log2(2^−ddCt^), mimic miR-146a-5p: 3.3 ± 1.0 log2(2^−ddCt^), *p* = 0.0495, Figure 6a). The same observation was made in THP1 cells, as transfecting cells with the miR-146a-5p mimic also led to reduced miR-146a-3p expression compared to NC (NC: 6.6 ± 1.0 log2(2^−ddCt^), mimic miR-146a-5p: 3.9 ± 0.4 log2(2^−ddCt^), *p* = 0.046, Figure 6b). No such effect was observed in HL60 cells, as only basal levels of miR-146a-3p were present (Figure 6c). Further, no influence of miR-146a-3p mimic transfection on miR-146a-5p expression was evident in A549, THP1, and HL60 cells (Figure 6d–f).

### 2.7. Expression of miR-146a-5p and miR-146a-3p Is Dysregulated in Inflammatory Lung Diseases

Since miR-146a-5p and miR-146a-3p have been found to be key players in our experimental model of inflammation, we next investigated their expression in existing NGS miRNA datasets from patients with inflammatory lung diseases such as COVID-19, CF, and PiBO.

**Figure 7:**
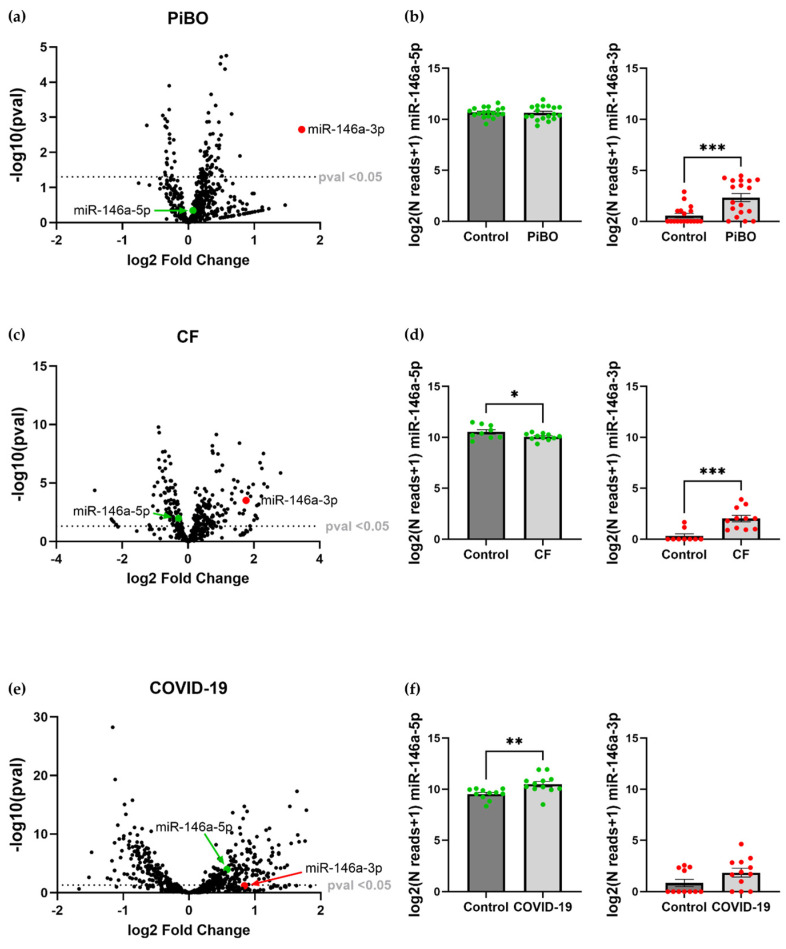
The expression of miR-146a-5p and -3p in representative inflammatory lung diseases. Volcano plots illustrating the expression of miR-146a-5p (green dot) and miR-146a-3p (red dot) analyzed by NGS in the serum of bronchiolitis obliterans (PiBO) (**a**), cystic fibrosis (CF) (**c**), and COVID-19 patients (**e**) compared to healthy controls. In bar graphs next to the volcano plots, the log2 normalized read counts+1 of the miR-146a-5p and miR-146a-3p expression of PiBO (**b**), CF (**d**), and COVID-19 (**f**) patients and controls are depicted with green and red dots representing patients or controls. PiBO: patients *n* = 19, controls *n* = 18; CF: patients *n* = 11, controls *n* = 9; COVID-19: patients *n* = 10, controls *n* = 11. * *p* < 0.05, ** *p* < 0.01, *** *p* < 0.001.

The volcano plots show that miR-146a-3p is significantly upregulated in the blood of patients with PiBO (log2 FC: 1.72, *p* = 0.002; Figure 7a) and with CF (log2 FC: 1.76, *p* = 0.0003; Figure 7c) compared to healthy controls, while miR-146a-5p expression is not significantly altered in PiBO and downregulated in CF (log2 FC: −0.29, *p* = 0.011; Figure 7c). This difference becomes particularly clear when the normalized miR-146a-3p reads of patients’ samples are compared (PiBO: 2.3 ± 0.4 log2(Nreads + 1), control: 0.6 ± 0.2 log2(Nreads + 1), *p* = 0.0004; Figure 7b; CF: 2.0 ± 0.3 log2(Nreads + 1), control: 0.3 ± 0.2 log2(Nreads + 1), *p* = 0.0005; Figure 7d). Further, normalized miR-146a-5p reads are significantly downregulated in CF patients compared to controls (CF: 10.1 ± 0.1 log2(Nreads + 1), control: 10.5 ± 0.2 log2(Nreads + 1), *p* = 0.0497; Figure 7d).

In contrast, in the blood of COVID-19 patients, miR-146a-5p is significantly upregulated (log2 FC: 0.6, *p* = 0.0001) whilst miR-146a-3p expression is not significantly elevated (Figure 7e). The comparison of the normalized reads revealed a significantly higher number of miR-146a-5p reads in the patients with COVID-19 compared to the control group (COVID-19: 10.5 ± 0.3 log2(Nreads + 1), control: 9.5 ± 0.2 log2(Nreads + 1), *p* = 0.006). There are no differences in the expression of miR-146a-3p (Figure 7f).

## 3. Discussion

Inflammation has an immense impact on the progression of various lung diseases with miR-146a posing as a promising novel target for its downregulation. In this study, we investigated the impact of miR-146a on the primary inflammatory cells implicated in respiratory diseases, namely lung epithelial cells, neutrophils, and macrophages, using a cell culture model to induce experimental inflammation. In contrast to previous studies, which focused on the anti-inflammatory role of miR-146a-5p, the aim of the present study was to investigate the dual role of the two isomiRs miR-146a-5p and miR-146a-3p on the modulation of inflammation on an individual basis as well as in combination.

Airway epithelial cells (A549), neutrophil-precursor cells (HL60), and macrophages (THP1) stimulated via the NF-kB pathway exhibited a major upregulation of the expression levels of miR-146a-5p and miR-146a-3p. Interestingly, miR-146a-5p was the only miRNA that was highly upregulated in all three cell types, followed by miR-146a-3p, which was significantly upregulated in A549 and THP1 cells. These findings are in line with other publications, identifying miR-146a-5p as a key regulator of inflammation, which is upregulated in A549 and THP1 upon the activation of the NF-kB pathway [27,30]. In HL60 cells, an increased expression of miR-146a-5p was reported, e.g., after phorbol 12-myristate 13- acetate (PMA) and hydroquinone treatment [45].

While the miR-146a-5p is well described in the literature, knowledge about the expression of the 3p arm of miR-146a in in vitro experiments is limited. A study stated that miR-146a-3p was the most highly upregulated miRNA expressed in A549 cells treated with RTHF, a traditional Chinese herb applied for its anti-cancer properties [46]. In addition, Gysler et al. found that the antiphospholipid antibody (aPL)-induced upregulation of trophoblast miR-146a-3p is mediated by TLR4 [40]. Less is known about miR-146a-3p expression in THP1 and HL60 cells. In our stimulation system, miR-146a-3p was found to be strongly upregulated in differentiated M1 THP1 cells and stimulated A549 cells but not in HL60 cells.

Besides miR-146a, another differentially expressed miRNA in stimulated A549 and HL60 cells was miR-3614-5p. Even though it was not included in our modulation experiments, miR-3614-5p plays an important role in inflammation. The expression of miR-3614-5p was increased after IFN stimulation in human immune and non-immune cells and is described as regulating the immune response by upregulating IFN-β and IL-6 [47]. Although miR-3614-5p was not significantly upregulated in the NGS analysis of THP1 cells, it was detected by qPCR in all three cell types as their stimulation included IFN-γ. A study by Huang et al. reported that miR-3614-5p is negatively regulating the release of inflammatory cytokines in THP1 cells by targeting *TRAF6* directly and consequently the NF-kB pathway [48]. The focus of our study comprised the analysis of miR-146a; however, miR-3614-5p could be included for future experiments to further elucidate its role during inflammation.

Of particular interest in our study was the analysis of the regulatory effects of the two single arms of miR-146a, as well as their combined modulation on inflammation using miRNA mimics and inhibitors. As described in other studies, we found an anti-inflammatory effect of miR-146a-5p overexpression [27,30]. miR-146 controls TLR and cytokine signaling through a negative feedback regulation loop, involving the downregulation of IRAK1 and TRAF6 protein levels [27]. In accordance with this, we demonstrated a significant downregulation of *IL-8* mRNA and protein expression in all stimulated cell lines. Vice versa, the application of anti-miR-146a-5p revealed reverse effects on IL-8 regulation, which confirms the existing knowledge about the anti-inflammatory properties of miR-146a-5p [27,30].

The overexpression of miR-146a-3p showed a significant pro-inflammatory effect, causing an increase in *IL-8* mRNA and protein expression. Notably, this effect was cell-specific, only detectable in lung epithelial and HL60 cells. This cell specificity of miR-146a-3p in inflammation is also reflected in the literature. While the administration of the miR-146a-3p mimic induced trophoblasts to secrete IL-8, miR-146a-3p transfection lead to a reduction in IL-17 expression in CD4+ T cells from the peripheral blood of children with neonatal sepsis [40,49]. The inhibition of miR-146a-3p demonstrated a robust reduction in IL-8 in A549, THP1, and HL60 cells on the protein level, pointing to the anti-inflammatory effect of blocking miR-146a-3p. Consistent with these findings, reduction in miR-146a-3p levels has been associated with beneficial effects, as it is reported to alleviate LPS-induced acute lung injury (ALI) in rats [35].

In two cases in HL60 cells as well as in THP1 cells, mimics and inhibitors of miR-146a-5p and miR-146a-3p, respectively, showed a similar effect on *IL-8* mRNA, downregulating its expression in both cases. These findings are contradictory to the expected outcomes of miRNA-target regulation and are hardly explainable. One possible approach could be distinct underlying mechanisms, which differ from the canonical target regulation by miRNAs. For example, a competition of the transfected miRNA inhibitor and the endogenous miRNA levels could impair the target gene regulation, leading to experimental bias [50,51]. Further, the target abundance can have an influence on the regulation by miRNAs. Lowly expressed target genes are stronger deregulated by miRNAs compared to highly expressed target genes [52]. However, these explanations are contrasting to the results observed in the other cell lines and transfection experiments even though a cell-specific distinct regulation mechanism is conceivable. More research is needed to confirm these results and exclude experimental bias to elucidate the underlying mechanisms.

However, the anti-inflammatory effects of miR-146a modulation were more distinctive at the protein than at the mRNA level. This indicates an imperfect match of miRNA-mRNA binding, leading to translational repression rather than mRNA degradation [53]. The underlying mechanism regarding why some effects were seen on the protein but not on the mRNA level is not yet elucidated; however, it is known that other post-translational processes influence protein expression. For instance, some miRNAs can interact with proteins beyond the canonical miRNA-mRNA regulation [54]. One example is TLR8, which can be directly activated by miR-146a-3p and thus induce the activation of NF-kB-mediated inflammation [40,54,55]. Other conceivable mechanisms involve nuclear activating miRNAs (NamiRNAs) and enhancer RNAs (eRNAs), which control transcription via interaction with promoters and via the activation of target genes [56]. However, more research is needed to clarify the underlying mechanism of the discrepancy between mRNA and protein expression.

Another aspect of the transfection experiments is that transfection with mimics was more efficient than with inhibitors, particularly observed for miR-146a-5p inhibitor transfection in our experiments. A possible explanation for this could be the fact that some endogenous miRNAs have weaker impact on highly expressed target genes. As a consequence, transfected, exogenous miRNAs possess stronger repressive functions for those targets, resulting in increased target deregulation by mimics and less efficient regulation by inhibitors [51]. To our knowledge, this is the first examination of the combined impact of miR-146a-5p and miR-146a-3p looking at cells implicated in respiratory diseases. Although we did not find an enhanced anti-inflammatory effect by applying the combination of the two miRNAs on IL-8 protein levels, the direct comparison of the anti-inflammatory properties of the two isomiRs showed a stronger effect of the inhibition of miR-146a-3p on IL-8 protein than the overexpression of miR-146a-5p. Moreover, while the inhibition or overexpression of one miRNA had no effect on inflammation in some cells, the inhibition of the other arm could significantly reduce inflammation. Similarly, in the publication of Gysler et al., it was demonstrated that miR-146a-3p has a functional role in aPL-induced IL-8 secretion in trophoblasts. Although the upregulation of both arms of miR-146a were detected in trophoblast exosomes, only the downregulation of miR-146a-3p showed an anti-inflammatory effect on IL-8 production, but not miR-146a-5p. Hence, the authors emphasize the importance of considering both arms of a promising miRNA [40].

In certain tissues, the co-expression of the 5p and the 3p arms of a miRNA is not uncommon as shown, e.g., for 19 miRNA pairs in colon cancer cells or specifically for miR-146 in thyroid cancer [57,58]. We also observed several pairs of co-expressed miRNAs in our study; e.g., both arms of miR-3614, miR-200a and -200b in A549 cells and miR-146b in HL60 cells were found to be dysregulated. The underlying mechanism of this phenomenon proposed by Choo et al. is a fail-proof system for gene regulation. Several miRNAs can target one gene and one miRNA may target different mRNAs of one biological process. Thus, the regulation can proceed unimpeded even if the integrity of one miRNA is impaired. The co-expression of 5p/3p miRNAs supports this system and can compensate the effect of, e.g., a mutation to maintain the biological function [57].

Being able to clearly demonstrate the co-expression of miR-146a-5p and miR-146a-3p in A549 and THP1 cells, our next step was to investigate if there is any mutual interference between the 5p and 3p arm. Indeed, we found that miR-146a-3p expression is downregulated by the addition of the miR-146a-5p mimic but not vice versa. This may demonstrate a dominance of the guide strand miR-146a-5p, conceivably as a body’s response to regulate inflammation, as enhanced miR-146a-3p expression levels were reported to correlate with disease severity [34,35]. As the upregulation of miR-146a-5p represents a natural control mechanism during inflammation by inducing the downregulation of the NF-kB pathway, this mechanism may be augmented by diminishing expression levels of the pro-inflammatory miR-146a-3p [23,59]. This effect could potentially be increased by simultaneously addressing both arms of miR-146a for impacting inflammation as observed in our study.

To finally transfer our observations of miR-146a in vitro with the expression during diseases in vivo, we re-analyzed existing miRNA expression data derived from patients with PiBO, CF, and COVID-19 as representative examples for inflammatory lung diseases. We found a significant miR-146a-5p upregulation in COVID-19 patients’ samples, which were obtained in an acute phase of COVID-19 disease. In contrast, we detected an upregulation of miR-146a-3p in conditions typically associated with chronic states, such as in the serum of patients with the chronic lung diseases PiBO and CF, whereas miR-146a-5p was not altered, indicating a disturbed resolution process in PiBO and CF. These findings are supported by Wang et al., who detected upregulated miR-146a-5p but not miR-146a-3p in an acute murine septic model [41]. In the literature, there are examples for a pro-inflammatory role of miR-146a-3p, as it is upregulated in exosomes from allergic rhinitis patients, which can be associated with disease severity [34], and the downregulation of the miRNA improves symptoms of ALI in rats [35]. However, a contrast is also reported: e.g., downregulated miR-146a-3p expression in patients with COVID-19 ARDS was shown to be correlated with the development of lung fibrosis [20]. In addition, it was demonstrated that the overexpression of miR-146a-3p attenuates airway inflammation in severe neutrophilic asthma by inhibiting Th17 cell differentiation [25]. These results suggest that miR-146a-5p and miR-146a-3p could potentially be regarded as biomarkers for chronic inflammatory processes. Certainly, this proposal must be validated in future research.

Strand selection plays a central role in post-transcriptional gene regulation, deciding which strand of the duplex miRNA will be loaded into the Argonaute protein and which will be degraded. There are many factors influencing the ratio of expression levels of 5p and 3p miRNAs. The relative thermodynamic stability and the 5’-nucleotide preference were identified as key factors for strand selection. Based on this knowledge, miR-146a-5p would be predicted as the guide strand. However, as reported by Medley et al., half of all miRNAs do not follow these rules [60].

It is further well established that the guide strand is usually more frequently observed compared to its counterpart, the passenger strand, though the relative expression of certain miRNA duplexes appears to be variable in different tissues [60]. For example, the 5p strand can be predominantly expressed in tumor tissue whereas the 3p arm is more prominent in healthy tissue [61]. Our results showed a cell-specific regulation of miR-146a-5p as well as of miR-146a-3p in all analyzed stimulated cell types. While miR-146a-5p is strongly represented in A549 cells, miR-146a-3p was slightly more expressed in THP1 cells and no miR-146a-3p was detected in HL60 cells.

The data give strong evidence that the modulation of the expression of both miRNAs had significant effects on IL-8 expression, indicating a functional role of both miRNA strands. In fact, double-stranded activity has already been described for several miRNAs such as miR-34a or miR-193b [62,63]. In these cases, both miRNAs were candidates for the Argonaute protein. On the other hand, an activating effect of miR-146a-3p is postulated by directly binding to the exosomal TLR8, indicating an alternative target regulation mechanism [40]. Furthermore, it is known that the presence of a specific target can impact the ratio of miRNA strands and even lead to their decay. This process is called “targeted–directed miRNA degradation” (TDMD) [64]. These examples illustrate the complexity of miRNA regulation and the importance of including both arms of a miRNA into any consideration.

To identify further targets of miR-146a-5p and miR-146a-3p, relevant pathways and targets were detected by an in silico analysis, showing targets implicated in TLR signaling, RIG-I-like signaling, and chemokine signaling. Modification experiments showed a regulatory role of both miRNAs for *IRAK1* and *TRAF6* expression, confirming knowledge from the literature [27,30,65]. Even though *IL8, IRAK1*, and *TRAF6* are listed as exclusive targets of miR-146a-5p in our in silico analysis, it was shown that miR-146a-3p is also a regulator of these target mRNAs [65]. *DDX3X* and *RNF215*, in contrast, are exclusively regulated by miR-146a-3p, showing decreased expression after anti-miR-146a-3p transfection. *DDX3X*, a member of the DEAD-box RNA helicase family, is described as enhancing inflammation by phosphorylating PP2A, an upstream target of the NF-kB signaling pathway [66]. Further, the downregulation of DDX3X is associated with protective effects against the development of sepsis in mice [67]. This implicates a possible involvement of *DDX3X* and consequently the RIG-I-like receptor signaling pathway in the regulation of inflammation by miR-146a-3p. However, *RNF125*, another member of the RIG-I-like receptor signaling pathway, is a negative regulator of RIG-I signaling and its downregulation is associated with increased release of pro-inflammatory mediators [68,69]. *CXCR4*, implicated in the chemokine receptor signaling pathway, is described as a target of both strands of miR-146a as confirmed by our experiments [70,71]. The downregulation of *RNF125* and upregulation of *CXCR4* consequently do not coincide with the anti-inflammatory effect observed by the overexpression of miR-146a-5p and the inhibition of miR-146a-3p in our experiments. However, only mRNA levels of specific miRNA targets were measured here, and the analysis of protein levels could give further conclusions about their involvement in inflammation regulation by miR-146a-3p.

Based on the significant role of both, miR-146a-5p and miR-146a-3p, in inflammation regulation, we speculate that a combined treatment addressing both arms of miR-146a may offer a beneficial avenue for novel approaches to treat chronic inflammation. This effect has been previously demonstrated by Zhang et al., stating that miR-574-5p and miR-574-3p imbalance is present in gastric cancer and that the two arms have antagonistic functions and targets in disease progression. They proposed a combination of inhibiting the 5p arm and overexpressing the 3p arm could be a promising new approach for treating gastric cancer [72].

However, this hypothesis warrants testing in future experiments, such as employing a mouse model to analyze specific targets and potential adverse effects following the combined modulation of miR-146a-5p and miR-146a-3p. A limitation of our study might be that inflammation was mainly assessed by measuring IL-6 and IL-8 levels, only including upstream targets of miR-146a such as *IRAK1, TRAF6, DDX3X, RNF125*, and *CXCR4* mRNA in A549 cells. Further, measurement of Argonaute–miRNA interaction could indicate if both miRNAs interact with the Argonaute protein in the canonical way or if an alternative mechanism of target regulation could be involved. Until today, a major obstacle in miRNA therapy was represented by off-target effects or unwanted on-target effects caused by the fact that miRNAs address multiple genes [73,74]. Indeed, such effects are directed in cancer therapy by combining low doses of synergistic miRNAs [75]. Nonetheless, a precise pathway analysis and a whole genome sequencing should be included in future research to exclude adverse effects of miRNA modulation.

## 4. Materials and Methods

### 4.1. Cell Lines and Stimulation of Cells

Human lung alveolar epithelial cell line A549 was obtained from DSMZ and was maintained in a DMEM medium (ThermoFisher, Dreieich, Germany) containing 10% fetal calf serum (FCS) (Sigma-Aldrich, Taufkirchen, Germany) and 1% penicillin–streptomycin (Life Technologies, Darmstadt, Germany) at a density of 2 × 10^6^ cells per T75 cell culture flask. For experiments, cells were seeded in 6-well plates with 5 × 10^5^ cells per well and were detached by using an accutase^TM^ cell isolation solution (Sigma-Aldrich, Taufkirchen, Germany).

The HL60 cell line, representing human promyelocytic cells/neutrophils, was purchased from DSMZ and was cultured in an RPMI 1640 medium + Glutamax (ThermoFisher, Dreieich, Germany) supplemented with 20% FCS and 1% penicillin–streptomycin. The THP1 monocytic cell line was provided by Prof. Dr. Dirk Henrich and was maintained in an RPMI 1640 medium + Glutamax plus 10% FCS and 1% penicillin–streptomycin. Both suspension cell lines were maintained in culture at a density below 1 × 10^6^ cells/mL and were used for seeding in 6-well plates with 1 × 10^6^ cells per well. All cell lines were grown in a humidified air incubator at 37 °C containing 5% CO_2_.

A549 and HL60 cells were stimulated for experiments for 24 h with a cytokine mixture (CM) containing different concentrations of IL-1β, IFN-γ, and TNF-α (PeproTech, Hamburg, Germany). The concentrations for the experiments were the following: CM4 with 0.5 U/mL IL-1β, 4 U/mL IFN-γ, and 0.2 ng/mL TNF-α; CM6 with 5 U/mL IL-1β, 40 U/mL IFN-γ, and 2 ng/mL TNF-α; and CM8 with 50 U/mL IL-1β, 400 U/mL IFN-γ, and 20 ng/mL TNF-α. THP1 cells were differentiated into M1 macrophages according to the protocol of Baxter et al., comprising a priming step using 5 ng/mL Phorbol 12-myristate 13- acetate (PMA) (Sigma-Aldrich, Taufkirchen, Germany) followed by a resting day in the cell culture medium and three days of polarization with 100 ng/mL LPS (Sigma-Aldrich, Taufkirchen, Germany) and 20 ng/mL IFN-γ [76].

### 4.2. miRNA Transfection

All cells were transfected using miRVana^TM^ Inhibitor anti-hsa-miR-146a-5p, anti-hsa-miR-146a-3p, hsa-miR-146a-5p miRVana^TM^ Mimic, hsa-miR-146a-3p miRVana^TM^ Mimic, or miRVana^TM^ miRNA Mimic Negative Control (ThermoFisher, Dreieich, Germany). For the determination of efficient transfection, different concentrations of miRVana^TM^ miRNA Inhibitor let-7c positive control and miRVana^TM^ miRNA Mimic miR-1 positive control (ThermoFisher, Dreieich, Germany) were tested in the cell culture system in each cell line. Results were analyzed by qPCR detecting mRNA levels of known targets of the miRNAs, namely High Mobility Group AT-Hook2 (*HMGA2*) and twinfilin 1 (*TWF1*), respectively. As transfection efficiency varies between each cell line, the optimal concentration and transfection time were tested individually and this was conducted as follows: A549 and THP1 cells were transfected with miRNA diluted with Opti-MEM media (ThermoFisher, Dreieich, Germany) and mixed with Lipofectamine RNAiMAX (1:1 *v*/*v*; Invitrogen, Karlsruhe, Germany) according to the manufacturer’s instruction (also see Fußbroich et al. [43]). For A549 cells, a concentration of 20 nM of miRVana^TM^ Mimic or Inhibitor was applied for 48 h. Differentiated THP1 cells were transfected with 40 nM Mimic or Inhibitor. Efficient transfection was analyzed after 24 h.

For the transfection of HL60 cells, SF Cell Line 4D-Nucleofector^TM^ X Kit S (Lonza, Cologne, Germany) was used, transfecting 750 nM miRVana^TM^ Inhibitor and Mimic with the Nucleofector X Unit according to the manufacturer’s instruction. Briefly, the cell pellet was resuspended in 4D-Nucleofector Solution, miRNA was added, and 20 µL of the cell–miRNA solution was pipetted per nucleocuvette. Cells were immediately transfected by program EN-138 in the nucleofector and incubated for 10 min at RT. An 80 µL pre-warmed RPMI medium was added and after another 10 min, cells were resuspended in a 140 µL cell culture medium in a 96-well plate. After 24 h post-transfection, HL60 cells were stimulated for another 24 h, and after a total of 48 h of incubation time, miRNA including total RNA was isolated for the analysis.

### 4.3. miRNA Isolation, Reverse Transcription, and Validation by qPCR

For the isolation of miRNA, including total RNA, the miRNeasy Mini Kit (Qiagen, Hilden, Germany) was used according to manufacturer’s instructions. Harvested cell culture samples were lysed in QIAzol Lysis Reagent (Qiagen, Hilden, Germany). Chloroform was added to the cell suspension and after centrifugation, the upper aqueous phase was mixed with 100% ethanol for the precipitation of RNA. RNeasy mini spin columns were used for washing and finally the elution of RNA in RNase-free water. To determine the RNA concentration and purity, samples were measured by a NanoDrop ND-1000 spectrophotometer (NanoDrop Technologies, Wilmington, DE, USA). Isolated RNA was stored at −80 °C until further use.

miRNA was transcribed to cDNA by using the TaqMan™ Advanced miRNA cDNA Synthesis Kit (ThermoFisher, Dreieich, Germany). Briefly, 5 ng of isolated RNA, including miRNA, was extended at the 3’ end of mature transcripts by the poly (A) tailing reaction. At the 5’ end, adapters were ligated, samples were reversely transcribed into cDNA using universal primers, and finally miRNA was amplified. A thermal cycler (GeneAmp Cycler PCR Systems 9700 v3.12., Thermo Fisher, Dreieich, Germany) was used for the reactions and programmed according to the manufacturer’s instructions. For the detection of miRNAs by qPCR, diluted cDNA (1:10 *v*:*v* in 0.1× Tris/EDTA buffer) was mixed with TaqMan™ Fast Advanced Master Mix and TaqMan™ Advanced miRNA Assay (has-miR-146a-5p, has-miR-146a-3p, has-miR-3614-5p; see Supplementary Material) (ThermoFisher, Dreieich, Germany) and measured in a Quant Studio 3 Real-Time PCR system (ThermoFisher, Dreieich, Germany). Results were analyzed by the 2^−ddCt^ method using *SNORD48* as the endogenous control.

### 4.4. Reverse Transcription and qPCR of RNA Samples

For processing total RNA, it was transcribed into cDNA by using the QuantiTect™ Reverse Transcription Kit (Qiagen, Hilden, Germany). At first, gDNA was removed by a gDNA wipeout buffer. All thermal cycler reactions were programmed according to the manufacturer’s instructions. A total of 2.5 ng/µL cDNA per sample was applied for qPCR using TaqMan™ Fast Advanced Master Mix and TaqMan™ Primer (ThermoFisher, Dreieich, Germany). Quant Studio 3 was used for PCR reactions and *GAPDH* served as an endogenous control for analyzing the samples by the 2^−ddCt^ method.

### 4.5. Library Preparation, NGS, and Data Analysis

Isolated miRNA samples were processed for library creation by using the QIAseq miRNA Library Kit (Qiagen, Hilden, Germany) and sequenced with miSeq Reagent Kit v3 and PhiX Sequencing control v3 (Illumina Inc., San Diego, CA, USA) as previously described by Duecker et al. [24]. A MiSeq Sequencer (Illumina Inc., San Diego, CA, USA) was used for sequencing and data were analyzed with the Qiagen GeneGlobe Data Analysis Center (https://geneqlobe.qiagen.com/de/analyze (accessed on 30 August 2023)). For quality control and differential expression, R version 4.2.0 (2022-04-22) was used. The DESeq2 method was applied for the comparison of raw read counts between two groups, and Fdr correction (Benjamini–Hochberg) was used for miRNAs passing DESeq-quality thresholds. miRNAs with padj < 0.05 were considered differentially expressed and those displaying padj < 0.001 were used for the further analysis.

### 4.6. Flow Cytometry

Cells were harvested, washed, and stained with antibodies (CD40, TLR4 (BioLegend, San Diego, CA, USA), CD11b, CD69 (ImmunoTools, Friesoythe, Germany), CD80 (ImmunoTech, Sofia, Bulgaria), CD86 (BD Bioscienes-Pharmingen, San Diego, CA, USA)) for 20 min in the dark. Cell surface markers were measured by a FACS Verse cytometer (BD Bioscienes-Pharmingen, San Diego, CA, USA) and 10,000 events were acquired and analyzed with FACS Suite software v1.0.6 (BD Biosciences-Pharmingen, San Diego, CA, USA).

### 4.7. Cytometric Bead Array

Inflammatory cytokines were measured in cell culture supernatants by a cytometric bead array (CBA) as described previously [77]. A BD FACS Verse cytometer (BD Biosciences-Pharmingen, San Diego, CA, USA) was used for measuring the samples, which were analyzed afterwards by FCAP Array Software v3 (BD Biosciences-Pharmingen, San Diego, CA, USA).

### 4.8. Protein Isolation and Western Blot

For the isolation of nuclear and cytoplasmic proteins, harvested cells were washed with ice-cold PBS and resuspended in an RSB buffer (10 mM Tris/HCl, pH 7.4, 10 mM NaCl, 3 mM MgCl_2_) supplemented with 0.5% Triton X 100. After lysing the cells for 5 min on ice, samples were centrifuged, and cytoplasmic protein was obtained in the supernatant. The pellets were washed with the RSB buffer and were subsequently resuspended in the RSB buffer containing 0.5 M NaCl for 40 min for lysing the cell nuclei. After centrifugation, nuclear proteins were collected in the supernatants and protein concentrations were determined using a NanoDrop ND-1000 spectrophotometer (NanoDrop Technologies, Wilmington, DE, USA).

A total of 20 µg protein of each sample was loaded onto a 10% SDS polyacrylamide gel. After electrophoresis, the gel was transferred onto a nitrocellulose membrane using a wet blot. Then, the membrane was blocked by 5% milk powder and incubated for at least 2 h with the corresponding primary antibodies (mouse anti-NF-kB p50 (Invitrogen, Karlsruhe, Germany), rabbit anti-YY1 (Abcam, Cambridge, UK)). After a washing step, the secondary antibodies (goat anti-mouse (Abcam, Cambridge, UK), donkey anti-rabbit (Invitrogen, Karlsruhe, Germany)) incubated again for at least 2 h followed by another washing step. The Western Lightning™ Plus-ECL Kit (PerkinElmer, Inc., Waltham USA) was used for the detection of protein bands, which were measured by a ChemiDoc^TM^ XRS+ System (Bio-Rad, Hercules, CA, USA).

### 4.9. Patients and miRNA Expression

miRNA data from PiBO, CF, and COVID-19 patients were re-analyzed from different studies as described in “Section 4.5”. More details about the patients’ characteristics can be found in [24,26]. NGS data from eleven patients with CF compared to nine healthy controls were included in the miRNA analysis. This study was approved by the Ethics Committee of Goethe University Frankfurt (E 13/18).

### 4.10. Pathway Enrichment Analysis of Target Genes

The Kyoto Encyclopedia of Genes and Genomes (KEGG) pathway analysis was performed using miRNet v2.0 (www.miRNet.ca (accessed on 27 June 2024)) and genes from the database miRTarBase v8.0. To identify pathways associated with inflammation, target genes of miR-146a-5p and miR-146a-3p were analyzed and pathways were manually chosen according to their padj values and their relevance.

### 4.11. Data Analysis and Statistics

Experimental data were analyzed and depicted with GraphPad v10.1.2 (GraphPad software, La Jolla, CA, USA) and R v4.2.0 (2022-04-22). qPCR expression levels of miRNAs and target genes were calculated as 2^−ddCt^ in relation to the endogenous control. To further illustrate the up- and downregulation of miRNAs/target genes in relation to the control, the data were log2-transformed.

For the comparison of two groups, an unpaired two-tailed *t* test was used. Results in the figures are depicted as the mean ± SEM and differences are regarded as significant with *p* < 0.05.

## 5. Conclusions

The miRNA-146a family plays a central role in inflammatory diseases. miR-146a-5p and miR-146a-3p are two distinct but related miRNA isoforms with opposing functions in the regulation of inflammation. Understanding their interaction provides important insights into the progression and persistence of inflammatory lung diseases and could offer potential therapeutic options.

## Data Availability

The data presented in this study are available on request from the corresponding author. The data are not publicly available due to privacy and ethical concerns.

## Supplementary Materials

The following supporting information can be downloaded at: https://www.mdpi.com/article/10.3390/ijms25147686/s1.
