# Supplementary material for: Dual Role of microRNA-146a in Experimental Inflammation in Human Pulmonary Epithelial and Immune Cells and Expression in Inflammatory Lung Diseases
Source: Int J Mol Sci. 2024 Jul 13;25(14):7686. doi: 10.3390/ijms25147686 (PMC11276706; doi:10.3390/ijms25147686)
Supplement: Supplementary file 1 [file ijms-25-07686-s001.zip › ijms-3014418-supplementary.pdf]

International Journal of
Molecular Sciences

Supplement Tables and Figures

Table S1: Dysregulated miRNAs in stimulated A549, THP1 and HL60 cells compared to unstimulated cells with padj <0.001 and FC >2 and < --2.

Cell line	Dysregulated miRNAs
A549	**Upregulated:**<br>hsa-miR-146a-5p<br>hsa-miR-147b<br>hsa-miR-205-5p<br>hsa-miR-146a-3p<br>hsa-miR-155-5p<br>hsa-miR-3614-5p<br>hsa-miR-3614-3p<br>hsa-miR-4515<br>hsa-miR-6501-5p<br>hsa-miR-6718-5p<br>hsa-miR-200a-3p<br>hsa-miR-455-5p<br>hsa-miR-455-3p<br>hsa-miR-200b-3p<br>hsa-miR-449a<br>hsa-miR-429<br>hsa-miR-200a-5p<br>hsa-miR-200b-5p<br>hsa-miR-548t-5p<br><br>**Downregulated:**<br>hsa-miR-222-5p<br>hsa-miR-558<br>hsa-miR-6747-5p<br>hsa-miR-654-3p<br>hsa-miR-5194<br>hsa-let-7c-3p<br>hsa-miR-1296-5p<br>hsa-miR-548v
THP1	**Upregulated:**<br>hsa-miR-184<br>hsa-miR-1-3p<br>hsa-miR-146a-5p<br>hsa-miR-146a-3p<br>hsa-miR-23b-3p<br>hsa-miR-548d-5p<br>hsa-miR-212-3p<br>hsa-miR-548a-3p<br><br>**Downregulated:**<br>-
HL60	**Upregulated:**<br>hsa-miR-3614-5p<br>hsa-miR-146b-3p<br>hsa-miR-146b-5p<br>hsa-miR-218-5p<br>hsa-miR-29b-1-5p<br>hsa-miR-146a-5p<br>hsa-miR-21-3p<br>hsa-miR-22-3p<br>hsa-miR-29a-5p<br><br>**Downregulated:**<br>-

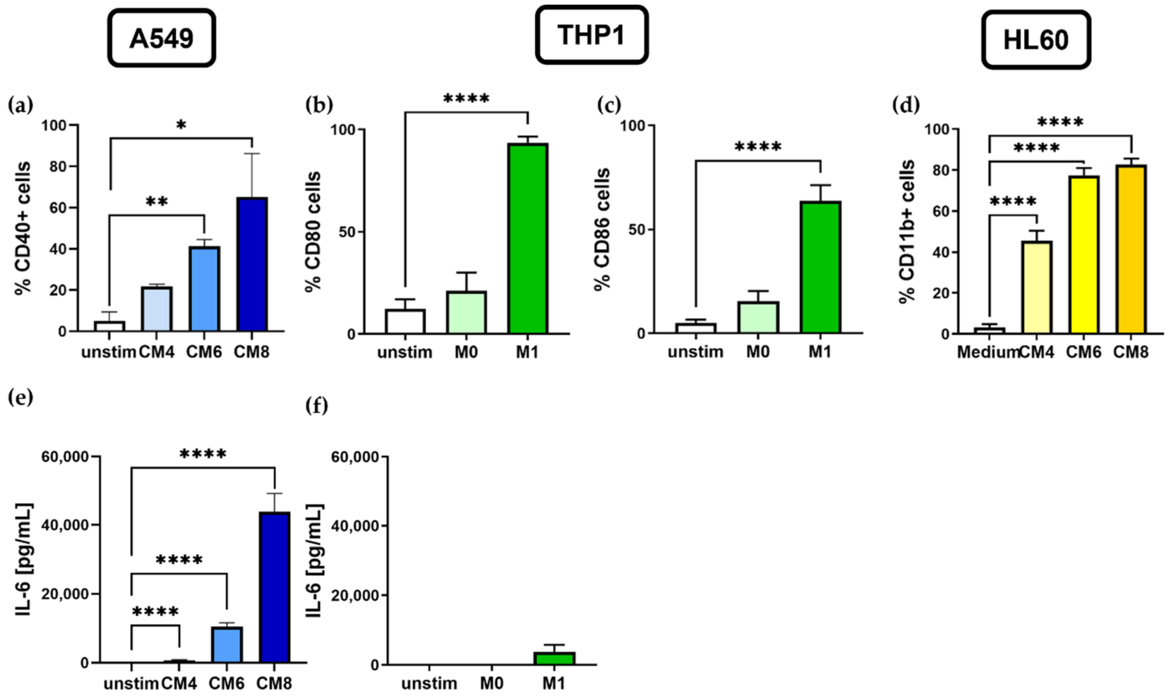

Figure S1: Expression of cell-specific markers after induction of experimental inflammation in vitro. CD40 (**a**), CD80 (**b**), CD86 (**c**) and CD11b (**d**) surface markers were measured by flow cytometry in stimulated A549 cells, THP1 and HL60 cells, respectively. IL-6 expression was measured by CBA in supernatants of A549 (**e**) and THP1 cells (**f**). A549: n = 3 – 8, THP1: n = 6, HL60: n = 5. * p <0.05, ** p <0.01, **** p< 0.0001

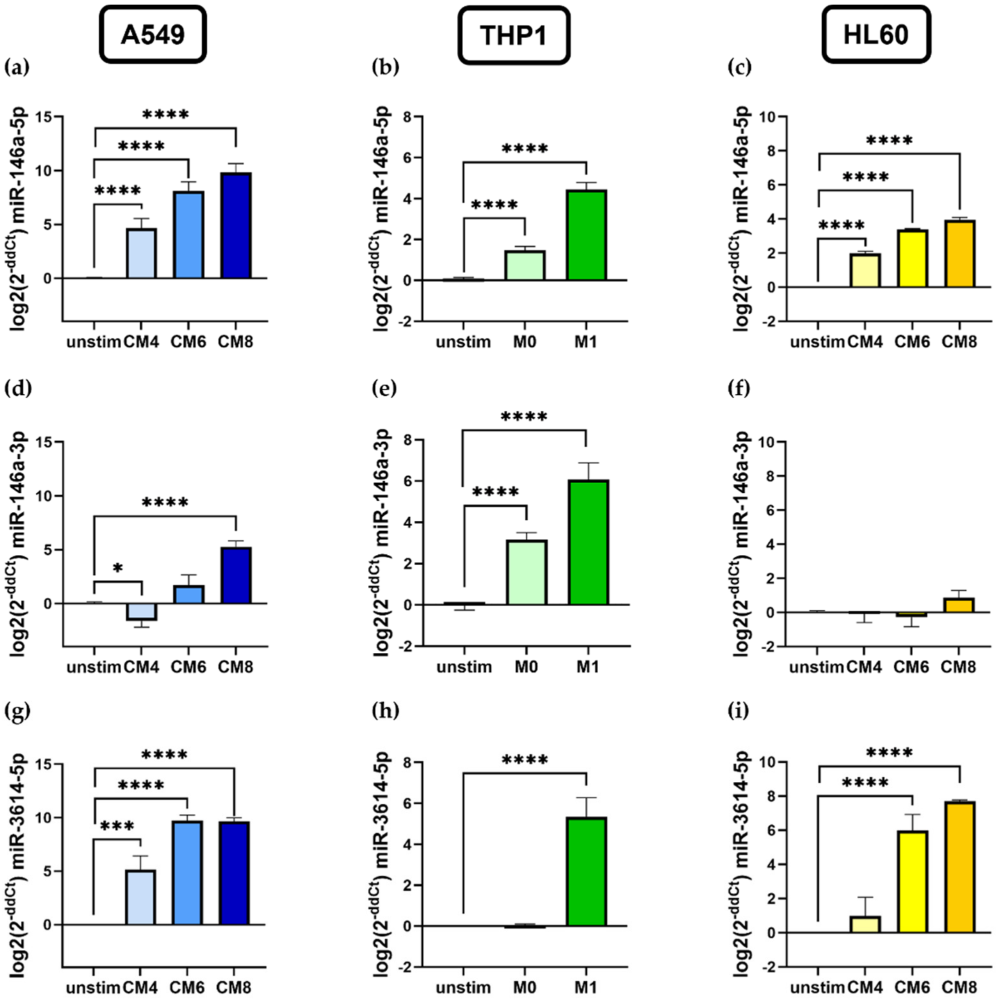

Figure S2: Validation of miRNA expression in stimulated cell lines. Expression of miR-146a-5p (**a-c**), miR-146a-3p (**d-f**) and miR-3614-5p (**g-i**) was validated by qPCR in stimulated A549, THP1, and in HL60 cells. A549: n =3-4, THP1: n = 3-6, HL60: n = 3-5. * $p < 0.05$, *** $p < 0.001$, **** $p < 0.0001$

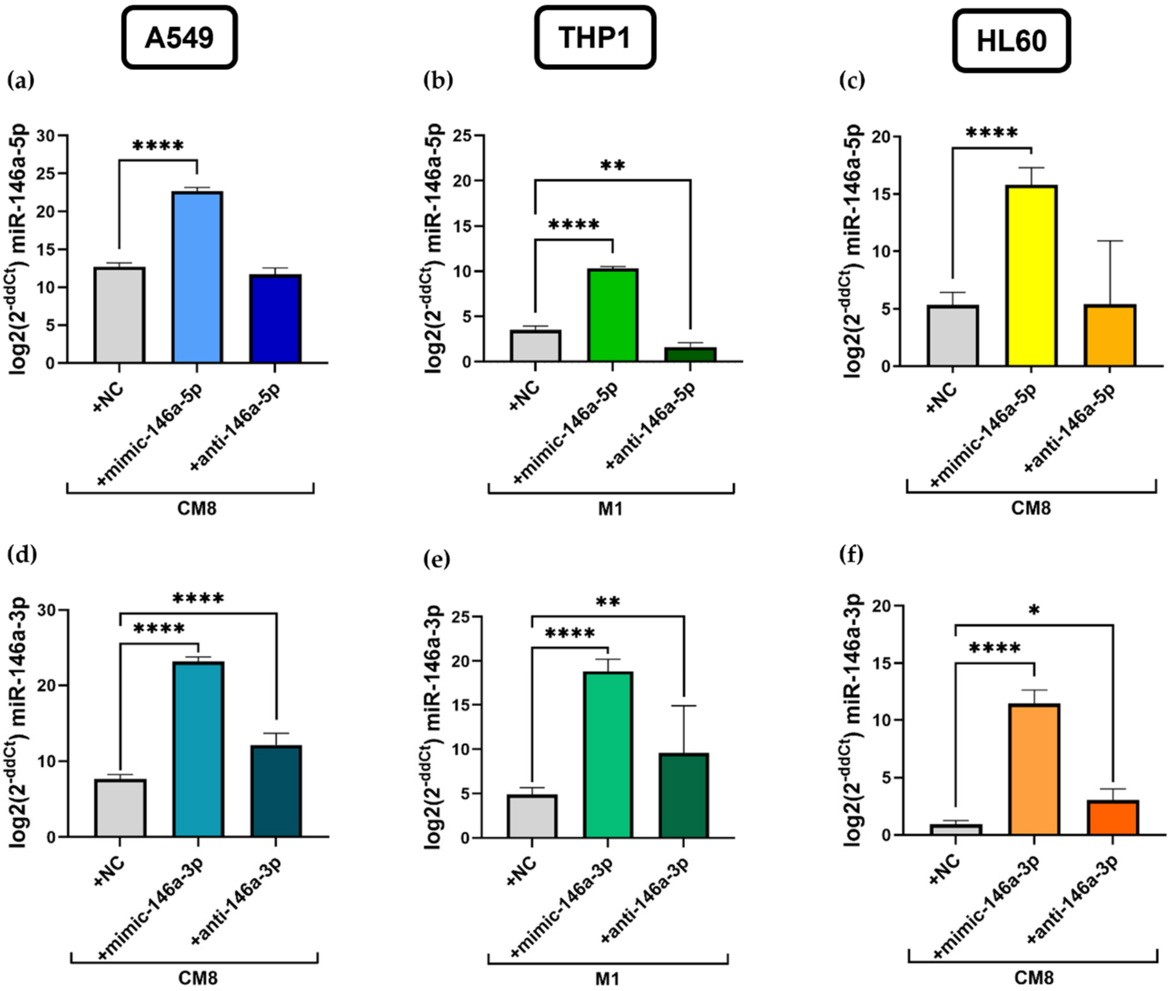

Figure S3: Transfection with miRNA mimics and inhibitors for in vitro modulation of miR-146a expression levels. Depicted are the expression levels of miR-146a-5p (**a-c**) and miR-146a-3p (**d-f**) measured by qPCR after transfection with respective mimics and inhibitors in CM8-stimulated A549, M1 THP1, and CM-8 stimulated HL60 cells. A549: n = 3 – 8, THP1: n = 6-7, HL60: n = 3-5. * p <0.05, ** p <0.01, **** p< 0.0001

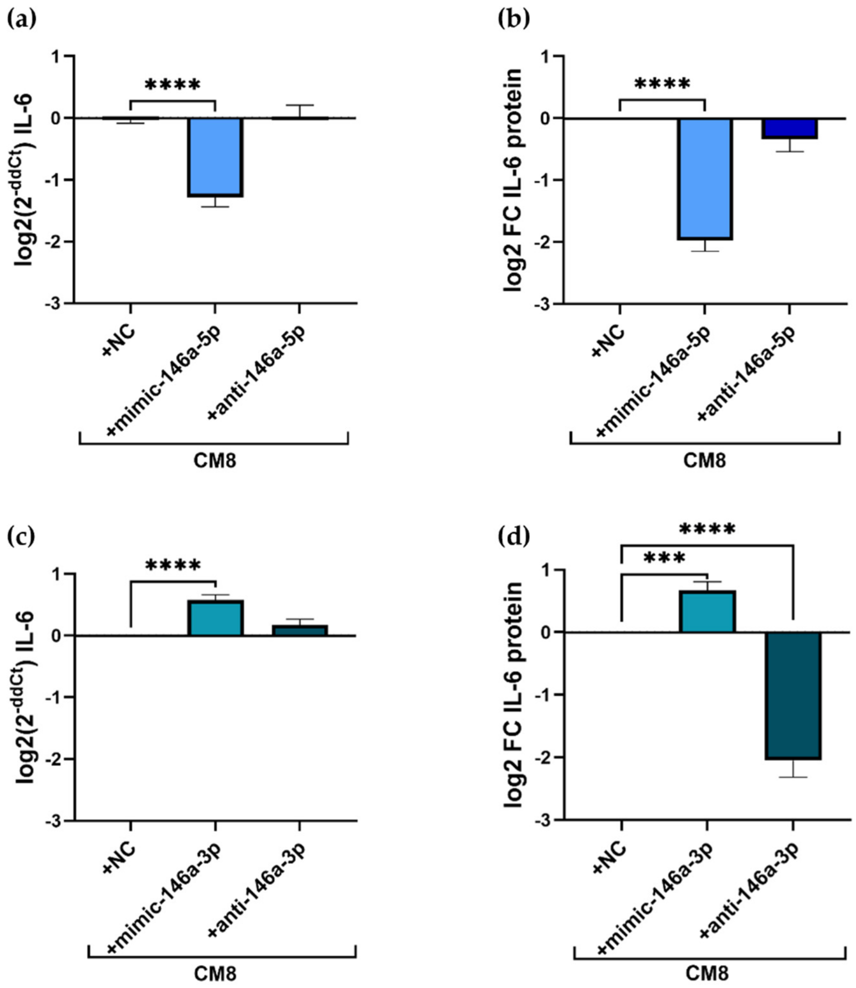

Figure S4: Effect of miR-146a-5p and -3p transfection on IL-6 expression in A549 cells. Bar graphs illustrating IL-6 mRNA (**a, c**) and protein expression (**b, d**) in CM8-stimulated A549 cells. Cells were transfected with mimic- and anti-miR-146a-5p (**a, b**) or with the mimic and inhibitor of miR-146a-3p (**c, d**). n = 7–12.*** p <0.001, **** p< 0.0001

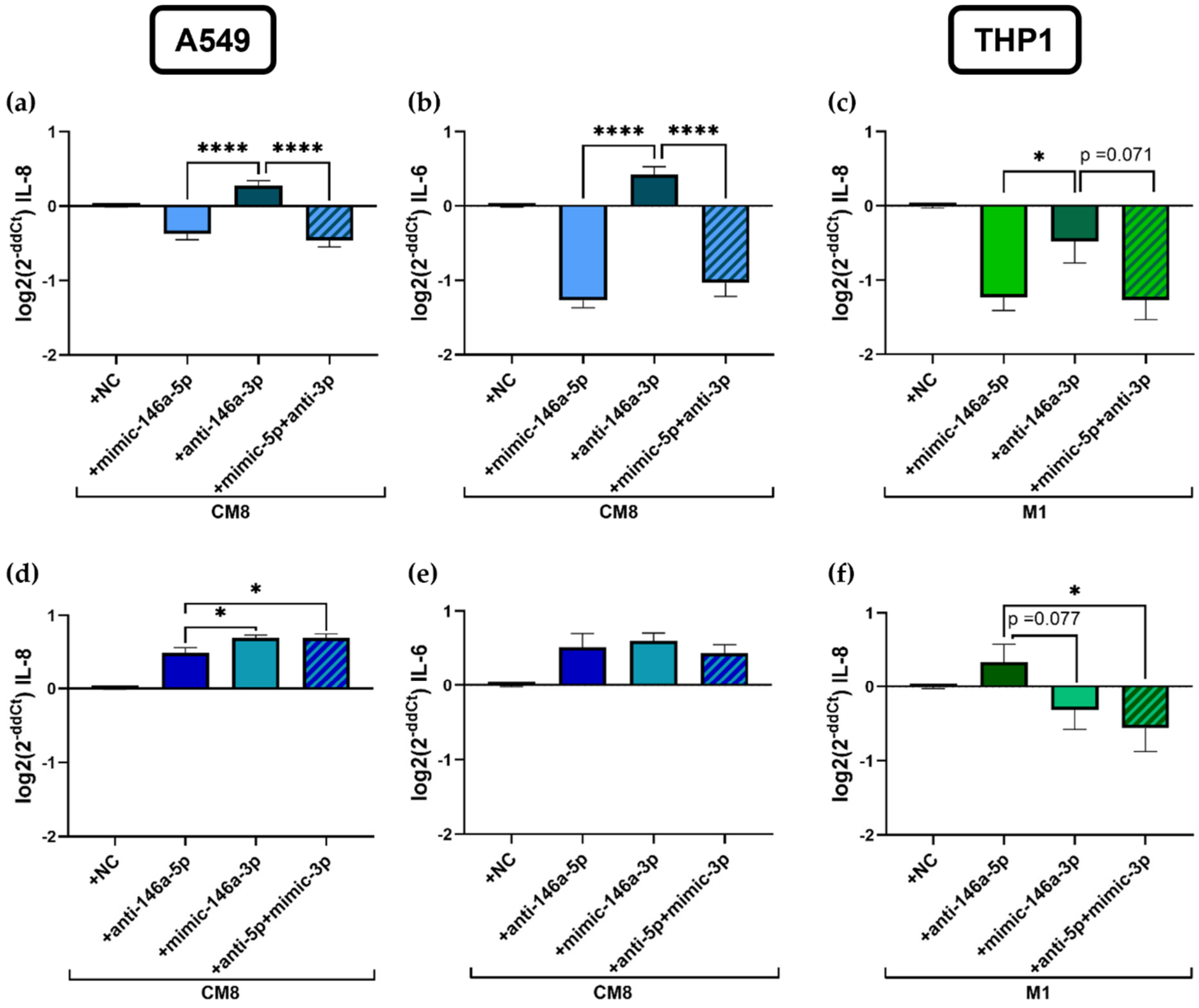

Figure S5: Simultaneous modulation of miR-146a-5p and 3p. In this figure, the mRNA expression measured by qPCR of *IL-8* in A549 (**a, d**) and THP1 cells (**c, f**) is shown. In A549 cells, additionally *IL-6* mRNA was detected (**b, e**). miR-146a-5p mimic and anti-miR-146a-3p were transfected individually and in combination in stimulated A549 cells (**a, b, d, e**) and THP1 cells (**c, f**). In graphs **d, e**, and **f** the anti-miR-146a-5p and miR-146a-3p mimic were applied single and in combination. A549: n = 5, THP1: n = 7. * $p < 0.05$, **** $p < 0.0001$

Supplement Material

In the following table all used primers are listed.

Primer	Mature miRNA Sequence	Assay ID	Manufacturer
CXCL8	-	Hs00174103_m1	ThermoFisher
IL-6	-	Hs00174131_m1	ThermoFisher
IRAK1	-	Hs00155570_m1	ThermoFisher
TRAF6	-	Hs00939742_g1	ThermoFisher
DDX3X	-	Hs00606179_m1	ThermoFisher
RNF125		Hs00215201_m1	ThermoFisher
CXCR4		Hs00607978_s1	ThermoFisher
GAPDH	-	Hs03929097_g1	ThermoFisher
SNORD48	-	Hs04931161_g1	ThermoFisher
Hsa-miR-146a-3p	CCUCUGAAAUUCAGUUCUUCAG	478714_mir	ThermoFisher
Hsa-miR-146a-5p	UGAGAACUGAAUUCCAUGGGUU	478399-mir	ThermoFisher
Hsa-miR-3614-5p	CCACUUGGAUCUGAAGGCUGCCC	478836_mir	ThermoFisher
